# Vascular Function in Chronic Non-Communicable Diseases

**DOI:** 10.3390/biomedicines10102520

**Published:** 2022-10-09

**Authors:** Manfredi Tesauro, Annalisa Noce

**Affiliations:** UOC of Internal Medicine-Center of Hypertension and Nephrology Unit, Department of Systems Medicine, University of Rome Tor Vergata, Via Montpellier 1, 00133 Rome, Italy

Chronic non-communicable diseases (CNCDs) are one of the major causes of mortality and morbidity worldwide [1]. This phenomenon continued to be relevant even during the pandemic, as CNCD patients were more susceptible to contracting infectious diseases, including severe acute respiratory syndrome coronavirus 2 (SARS-CoV-2)—but above all, they developed more severe pictures.

In the pre-pandemic era, a growing increase in CNCDs incidence was observed, related to the spread of obesity, unhealthy dietary habits and the lengthening average life span. Therefore, geriatric subjects seem to have a higher risk of developing CNCDs, and occasionally, they present multiple CNCDs at the same time [2,3,4].

The dysfunction of the endothelium and of the vascular system plays a pivotal role in the pathophysiology of CNCDs. This vascular impairment is induced and worsened by the increased production of reactive oxygen species (ROS) and an enhanced release of pro-inflammatory cytokines. A strong association between inflammation and oxidative stress has been demonstrated. In particular, in the site of inflammation, polymorphonuclear neutrophils produce ROS, inducing endothelium dysfunction and tissue injury [5]. At this regard, oxidative stress and low-grade chronic inflammatory status are peculiar features of CNCDs and they are involved in the progression of CNCDs. Physiologically, the ROS act as signaling molecules, and they regulate cellular growth and differentiation [6]. On the contrary, their chronical increased production is crucial for the evolution of CNCDs [7].

Among therapeutic strategies able to counteract vascular dysfunction and the progression of CDNCDs, we include moderate physical exercise, healthy dietary habits, and the use of drugs or oral food supplements that exert an endothelium-protective action [8,9,10].

In this Special Issue, we published some interesting original articles and reviews about this research field. In detail, Andújar-Ver et al. [11] evaluated a new link between the cardiovascular/Alzheimer’s axis through bioinformatics approaches. The authors correlated the presence of apolipoprotein E, haptoglobin, clusterin, and alpha-2-macroglobulin in both diseases, speculating on their key roles in these two pathological conditions.

Kim Y. et al. examined the possible influence of fibromyalgia on cardiometabolic complications, highlighting that women with fibromyalgia have a higher risk of developing central obesity, hypertriglyceridemia, impaired fasting glucose, and advanced arterial stiffness of the carotid artery compared to healthy controls [12].

Chen Z.W. et al. studied whether heart-ankle pulse wave velocity is superior to brachial-ankle pulse wave velocity in detecting aldosterone-induced arterial stiffness, demonstrating that the former was a more predictive parameter than the second one [13].

Van Den Hoven P. and co-authors examined perfusion patterns in chronic limb-threatening ischemia patients compared to healthy subjects through near-infrared fluorescence imaging with indocyanine green. They also pointed out how the first patients presented altered regulatory mechanisms of microcirculation and arterial stiffness [14].

An animal study by Sauvé M. F. investigated the impact of glycomacropeptide on insulin resistance and hepatic dysmetabolism. The authors concluded that glycomacropeptide supplementation for 12 weeks exerted important antioxidant and anti-inflammatory actions and improved insulin sensitivity [15].

Schinzari F. et al. monitored the correlation between an impaired vasodilator reactivity (an early abnormality in atherosclerosis) with the levels of the circulating angiopoietin-like-3 and -4 in obese patients, demonstrating that increased circulating angiopoietin-like-3 values were present only in metabolically unhealthy obese patients. On the contrary, the circulating angiopoietin-like-4 values were enhanced in all obese patients [16].

Moreover, Colombo G.I. and collaborator assessed the possible link between cholesteryl ester transfer protein (CETP) and carotid intima-media thickness. They also assessed the impact of high-density lipoprotein cholesterol (HDL-C) on this relationship in high-risk cardiovascular patients. The authors demonstrated a direct CETP-dependent correlation between HDL-C levels and carotid atherosclerosis [17].

Finally, this Special Issue includes two reviews: the first investigated the importance of evaluating the coexistence of coronary and peripheral disease in erectile dysfunction patients [18]. The second examined the potential beneficial effects of antidepressant therapies on the reduction of vascular inflammation and of arterial stiffness [19].

In summary, the nine papers published in this Special Issue highlighted the role of endothelial dysfunction in cardiometabolic diseases, also studying new possible biomarkers of vascular dysfunction.

## Abbreviations

CETP	Cholesteryl ester transfer protein
CNCDs	Chronic non-communicable diseases
HDL-C	High-density lipoprotein cholesterol
ROS	Reactive oxygen species
SARS-CoV-2	Severe acute respiratory syndrome coronavirus 2
